# Assessment of dermatological life quality in patients with cutaneous diseases at Universitas Academic Hospital

**DOI:** 10.4102/jcmsa.v3i1.160

**Published:** 2025-03-31

**Authors:** Navlin Naidoo, Frans Maruma, Edward Ngwenya, Mthobisi N. Mazibuko

**Affiliations:** 1 Department of Dermatology, Faculty of Health Sciences, University of the Free State, Bloemfontein, South Africa; 2 Department of Dermatology, Faculty of Health Science, University of Pretoria, Pretoria, South Africa; 3 Department of Dermatology, Faculty of Health Science, University of KwaZulu-Natal, Durban, South Africa

**Keywords:** dermatological life quality index, DLQI, dermatological diseases, psychometrics, quality of life, burden of skin diseases

## Abstract

**Background:**

Dermatological conditions significantly impact quality of life (QoL). This cross-sectional study aimed to assess this impact as part of a comprehensive care approach.

**Methods:**

A cross-sectional analytical study was conducted at a tertiary hospital’s dermatology outpatient department from January 2023 to March 2023. Eligible patients were recruited, and data on demographics, clinical diagnoses and Dermatological Life Quality Index (DLQI) scores were collected.

**Results:**

The study included 200 participants (median age: 39 years, range: 18–86). Females comprised 58%, while males were significantly older (median: 53 vs 35 years, *p* < 0.001). A significant difference was found between the 10 most common diagnoses and DLQI scores (*p* < 0.05, Kruskal–Wallis test). The top diagnoses were eczema (21%), cutaneous malignancy (13%) and acne/rosacea (11%). Hidradenitis suppurativa had the highest DLQI scores, followed by lupus and urticaria.

**Conclusion:**

This study highlights the significant burden of skin diseases on QoL, with hidradenitis suppurativa showing the greatest impact. Findings underscore the need for patient-centred dermatological care addressing both physical and psychological well-being.

**Contribution:**

This study contributes to dermatology research in line with the *Journal of the Colleges of Medicine of South Africa* (JCMSA) mission to encourage multidisciplinary publications.

## Introduction

The skin, as the largest and most visible organ in mammals, serves as a primary defence mechanism and plays a crucial role in physical appearance and interaction with the external environment.^1^ Chronic skin diseases can profoundly impact various aspects of an individual’s life, including emotional well-being (e.g., insomnia and anxiety), daily functioning and social relationships. Research indicates that the chronic nature of inflammatory dermatological conditions disrupts physical, social and psychological well-being, leading to a decline in health-related quality of life (HRQoL).^2^

The degree to which dermatological diseases negatively affect individuals depends on several factors: body surface area involved, disease activity, lesion location and symptoms like itching, discomfort and pain.^3,4^ For example, atopic eczema significantly impacts HRQoL, notably affecting mental health, social engagement and emotional stability. Studies have shown that disease intensity correlates with poorer QoL for both adults and children. The World Health Organization (WHO) defines QoL as an individual’s perception of their position in life, shaped by cultural and value systems and influenced by personal goals, expectations, standards and concerns.^5^ Numerous studies have evaluated QoL outcomes in patients with diseases like psoriasis, chronic pruritus and atopic dermatitis, emphasising the importance of incorporating DLQI assessments into patient management.^6,7,8^

Atopic dermatitis is one of the most cited skin diseases that affect QoL. The disease may persist into adulthood, imposing significant financial burdens because of high healthcare costs.^9^ In Japan, a study exploring the relationship between disease severity and productivity found a strong correlation between disease severity – measured using the Severity Scoring of Atopic Dermatitis (SCORAD) and DLQI – and reduced productivity and daily activity.^10^ Although many dermatological diseases are not life threatening, their chronic nature and impact on daily life are often underestimated.^11,12,13^

Globally, skin diseases affect a substantial portion of the population. McKoy’s study estimated that between 21% and 87% of people worldwide experience some form of dermatological illness.^13^ Assessing the economic burden of skin diseases is particularly challenging in developing countries, where access to dermatological care is often limited. Primary healthcare providers frequently face difficulties diagnosing and managing skin conditions effectively. Reports indicate that patients in these regions may spend 50%–100% of their income on treatments, which prove ineffective for up to 75% of cases.^13,14^ Furthermore, about 24% of patients visiting primary healthcare clinics for dermatological concerns seek treatment because of issues related to self-image, disfigurement or physical impairment.^13^

Even when physical impairment is minimal, the visible and sometimes disfiguring nature of skin diseases can significantly impact mental health, further affecting QoL.^14,15,16^ Beyond atopic dermatitis, conditions such as alopecia areata, psoriasis, hidradenitis suppurativa (HS) and pemphigus vulgaris have similarly severe effects. Hidradenitis suppurativa, for example, often causes malodour, leading to social stigmatisation and mental health struggles, as it is mistakenly associated with poor personal hygiene.^17,18,19,20,21^ For pemphigus vulgaris, there is a negative correlation between DLQI scores and disease duration^22^ Conditions like psoriasis and atopic dermatitis frequently cause psychological and social distress, with patients commonly experiencing depression and fear of stigma.^18,19^

Both psoriasis and atopic dermatitis have been extensively studied worldwide, and patients treated with biologic therapies like etanercept and infliximab often experience significant improvements in QoL. A Colombian study of over 1800 participants highlighted atopic dermatitis and psoriasis as major diagnoses that negatively impact QoL.^23,24^ Acne vulgaris also plays a role in diminished life quality, with research indicating that adolescents and young adults often suffer from lowered self-esteem because of their skin’s appearance, especially in the context of social media.^24,25,26,27^

### Rationale for the study

To our knowledge, no comprehensive studies in South Africa have examined the DLQI of patients across a variety of skin disorders in dermatology clinics. While some research has focused on the impact of specific skin conditions on QoL among different social and racial groups, none have collectively assessed DLQI for a broad spectrum of dermatological conditions.^28,29,30,31,32^ Universitas Academic Hospital is the only dermatology clinic that treats a diverse patient population mainly in the state sector as evidenced by a decade of patient registry data, except for the disruptions during the coronavirus disease 2019 (COVID-19) lockdown. Additionally, human immunodeficiency virus (HIV) and/or acquired immunodeficiency syndrome (AIDS) remain a significant burden on South Africa’s healthcare system, with various skin manifestations serving as markers for disease progression.^33,34,35^

This study thus aimed to assess how different skin disorders impact the patients’ QoL through achieving the objectives listed as follows. Primary Objective: To evaluate and compare the DLQI scores among patients diagnosed with different dermatologic conditions. Secondary Objective: To identify potential demographic or clinical factors that may influence quality of life outcomes in patients with dermatological conditions.

## Research methods and design

### Study design

This was a cross-sectional descriptive study that was conducted from January 2023 to March 2023.

### Setting

The study was conducted in the dermatology outpatient department of the tertiary Universitas Academic Hospital. This tertiary hospital is academically affiliated with the University of the Free State. Notably, it is the only state facility providing dermatological care and services in the state sector of the Free State province.

### Study population and sampling strategy

A calculated sample size of 200 patients was determined to achieve reliable and representative results for this study. The sampling method employed was systematic sampling, with randomisation introduced to minimise selection bias. Specifically, every alternate patient seen at the clinic was invited to participate in the study. By incorporating this structured randomisation, we aimed to provide an equal and unbiased opportunity for patient selection. The study population consisted of patients known to the dermatology clinic, with inclusion and exclusion criteria as set out below.

#### Inclusion criteria

- Patients aged 18 years and above.
- Diagnosed with one or more dermatological conditions for more than 3 months at the time of enrolment.
- Willing to participate and provide informed consent.

#### Exclusion criteria

- Patients unable to provide informed consent.
- Those with psychiatric conditions that may affect the assessment of QoL.

To maintain data quality and ensure participants’ ability to respond thoughtfully and accurately, patients with a history of psychiatric illness or those currently receiving psychiatric medication were excluded. This step was taken to ensure that all participants had normal mental functioning and could provide open, unbiased responses to the study questions.

### Data collection

Patients who were diagnosed with any dermatological conditions at outpatient clinic of the Universitas Academic hospital 3 months prior to the study, namely January 2023 to March 2023. Qualifying participants were considered according to the inclusion and exclusion criteria. A DLQI questionnaire (validated instrument used to assess the impact of skin conditions on QoL) was used to collect data from participants.

Further data collected included demographic variables and clinical information (diagnosis and its duration), in addition to data derived from completion of the DLQI questionnaire.

### Data analysis plan

The data were analysed in collaboration with a biostatistician. Data entry and management were performed using Microsoft Excel, and the dataset was subsequently imported into Statistical Package for Social Sciences (SPSS) version 29 for analysis. Statistical significance was determined at a 0.05 alpha level.

### Description of measures

The DLQI was calculated by summing the scores of individual items, with a potential range of 0–30. A higher DLQI score indicates greater impairment of the participant’s QoL.

### Statistical analysis

Analysis of data was performed to evaluate differences across demographic and clinical characteristics, using appropriate non-parametric statistical tests for skewed data distributions

### Categorical variables

- Comparisons between groups were conducted using either Fisher’s exact test or the Pearson Chi-squared test, based on the sample size and expected frequency distributions.

### Continuous variables

#### Median age and dermatological life quality index

The Mann–Whitney *U* test was applied to compare the median age and median DLQI scores between male and female participants.

#### Dermatological life quality index across diagnoses

The Kruskal–Wallis test was employed to compare the median DLQI scores among different diagnostic categories.

### Ethical considerations

The protocol was submitted to Health Sciences Research Ethics Committee (HSREC) for interrogation and processing. Ethical clearance to conduct this study was obtained from the University of the Free State Health Sciences Research Ethics Committee (No. UFS-HSD2022/1564/2803). No identifiable patient information was used and all patients gave informed consent prior to participating in this study.

## Results

**FIGURE 1:**
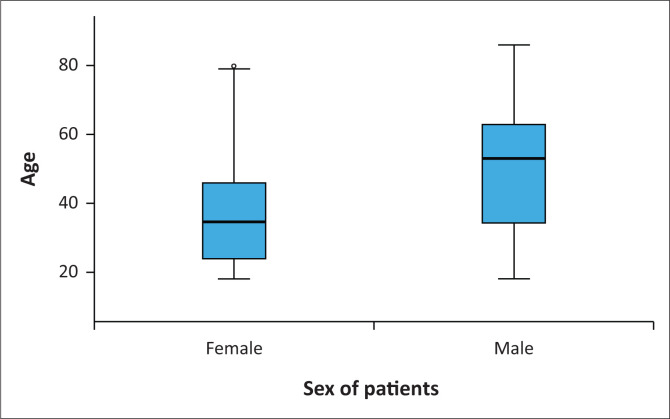
Participants’ characteristics.

**FIGURE 2:**
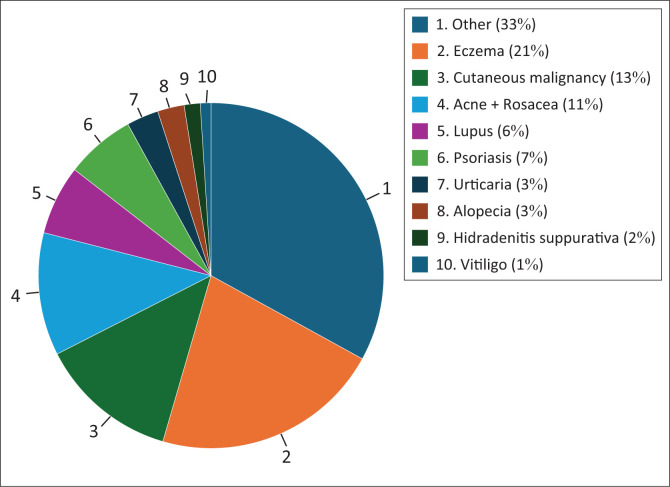
Frequency of diagnoses (*N* = 200).

Figure 1 demonstrates that males are generally having a wider age range and a higher median age compared to females. Of the 200 patients included, the results show that the top three conditions are eczema, rosacea + acne and cutaneous malignancies as illustrated in Figure 2.

Table 1 summarises the distribution of dermatological diagnoses by sex, and the Pearson’s Chi-square test that indicates that there is a statistically significant difference in distribution between females and males (*p* = 0.029). Table 1 further illustrates more female predilection, i.e., with eczema accounting 23%, Lupus, 10.3% and Acne + rosacea (12.1%).

**TABLE 1:** Association between diagnosis and sex.

Diagnosis	Sex of patients			
	Female		Male	
	*n*	%	*n*	%
Psoriasis	8	6.9	5	6.0
Eczema	27	23.3	16	19.0
Lupus	12	10.3	1	1.2
Acne + Rosacea	14	12.1	9	10.7
Urticaria	5	4.3	1	1.2
Cutaneous malignancies	10	8.6	16	19.0
Hidradenitis suppurativa	3	2.6	0	0.0
Alopecia	3	2.6	2	2.4
Vitiligo	0	0.0	2	2.4
Other	34	29.3	32	38.1

**Total**	**116**	**100.0**	**84**	**100.0**

Note: Pearson’s exact Chi-square = 18.57 *p* = 0.029

The highest median score of 18 was found in both lupus and HS, implying significant life quality impairment for these two chronic conditions. It is then followed by urticaria (DLQI = 10). The total median score for all the diagnoses is 7, indicating a moderate impairment of QoL (Table 2). Lupus formed part of the top three diagnosis as well as having the highest DLQI among all conditions.

**TABLE 2:** The interquartile ranges for dermatological life quality index scores per diagnoses.

Diagnosis	DLQI score		
	Median	Percentile 25	Percentile 75
Psoriasis	9	5	12
Eczema	8	3	14
Lupus	18	9	22
Acne + Rosacea	4	1	10
Urticaria	10	6	11
Cutaneous malignancy	5	1	10
Hidradenitis suppurativa	18	16	20
Alopecia	9	9	18
Vitiligo	8	8	8
Other	6	2	10

**Total**	**7**	**3**	**13**

DLQI, dermatological life quality index.

The Kruskal–Wallis test was used to assess the difference between DLQI scores across various groups. There is apparent variability in means suggesting that there could be significant differences, particularly between HS and vitiligo. However, one would have to perform post hoc pairwise tests such as Dunn’s test to identify which group differs significantly. This was, however, beyond the scope of this present study.

## Discussion

The sociodemographic characteristics of this study reflect that females appeared to be more significantly affected than males (Figure 1). There was also a statistically significant association between diagnosis and sex (*p* = 0.029) with malignancy and other conditions being higher in females (Table 1). However, it is important to consider that females constituted a higher proportion of the study sample (58%) compared to males. One possible explanation for this gender discrepancy could be that females are more likely to visit dermatological clinics, a trend also observed in other studies.^23,36^

Another noteworthy demographic finding was related to age. Specifically, younger participants, with a median age of 39 years, were more commonly affected by skin diseases. This observation aligns with existing research, which has also shown that the younger the age group, the more the likelihood of experiencing greater impairment in QoL because of skin conditions.^23,33,36^

This study also demonstrated that the QoL of patients with varying skin diseases was variably impacted. The top three frequent dermatological conditions presented among the participants were those of eczema (21%), cutaneous malignancies (13%) and acne + rosacea (11%) (Figure 2). Their respective DLQIs are 9, 5 and 4 (Table 2). Among all the dermatological conditions presented in this study, nine conditions were identified as the common conditions that impacted on the QoL of patients. These were HS cutaneous lupus, urticaria, alopecia, psoriasis, vitiligo, eczema, cutaneous malignancy and acne with HS impacting on the quality of a patient’s life the most. Of significant importance, other pigmentary disorders (e.g. melasma) other than vitiligo were not observed in this cohort. The main reason could be that the study site (state facility) exclusively deals with pathological dermatology rather than aesthetic concerns. Notably, 33% of dermatoses were classified as ‘other’ because they each fell into a wide and heterogeneous range of categories that could not be combined or classified into a single group or disease entity of significance (Figure 2).

Even within this miscellaneous group of disorders, one could still not contentedly single out a greater proportion that could account for facial dyschromia such as post-inflammatory pigmentation, ashy dermatosis, dermatosis papulosa nigra, melasma and Nevus of Hori as described in recent south African literature by Maruma et al.^37,38,40^

This study did not identify dyschromia’s among the top five dermatological conditions, which contrasts with findings from recent studies conducted in South Africa, wherein dyschromia’s were ranked prominently among the most common dermatological presentations^39,40^. This discrepancy suggests that factors such as socioeconomic status, healthcare accessibility and patient demographics may significantly influence disease prevalence in different settings such as the state versus private facilities. Furthermore, the facility where this study was conducted does not specifically cater to pigmentary disorders, which may have contributed to the observed distribution. These findings highlight the need for integrated approach and partnership between private and public health providers to work together in addressing topical issues within the fraternity. The epidemiological data obtained in the state facility are completely different from the private sector.^40^

The identified dermatological conditions that impacted the most in the present study correlate well with other QoL studies conducted in other countries. Urticaria and eczema were also among the top nine conditions affecting QoL of patients as cited in other studies.^14,41,42^

**FIGURE 3:**
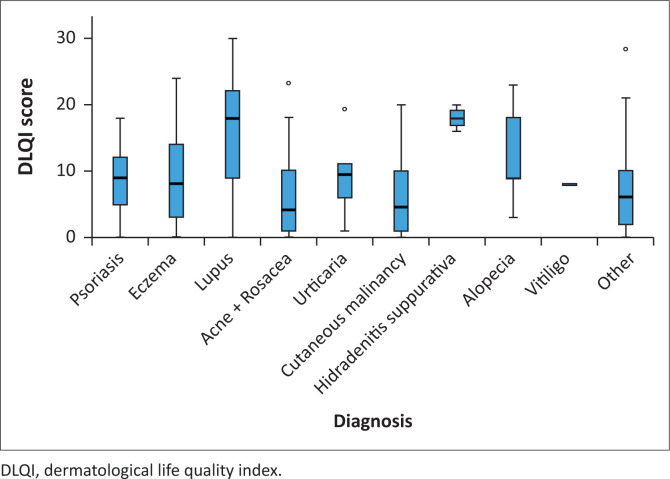
Asymptotic significance displayed for various diagnosis and dermatological life quality index scores.

By examining the relationship between the DLQI scores per diagnoses, we observed that HS (DLQI = 18) had the highest scores (most impairment) along with lupus (DLQI = 18) and followed by urticaria (DLQI = 10) (Table 2). Furthermore, we used the Kruskal–Wallis test to assess whether these various dermatologic conditions differ in their impact on QoL (Figure 3). The data revealed substantial variability in DLQI scores across these conditions with HS, lupus and urticaria exhibiting the highest median scores, indicating that these conditions were associated with a significant reduction in QoL (Figure 3). This implies that patients with these diagnoses may experience greater psychological, social and physical burdens.

Conversely, conditions such as acne + rosacea (DLQI-4) and eczema (DLQI-8) and others collectively had lower median DLQI scores; however, these entities each had a significant impact on QoL (Figure 3). Overall, the analysis highlights the need for targeted interventions for conditions with higher DLQI scores, such as HS and lupus, to improve patient outcomes. The nature of the disease severity, the topography (in acne+ or rosacea) HS in armpits, under breast and groin area, as well as symptomatology as in pruritus in urticaria, all invariably contributing to the poor QoL that these patients face. For example, HS can be oozing pus and debilitating to the patient while they are in intractable pain and have a malodour that other members of public would pick up and stigmatise. Therefore, the impairment of QoL has far-reaching and un-imaginable consequences.

Understanding the DLQI scores of patients could be instrumental in tailoring management approaches to offer more holistic and effective treatment plans. This could further provide patients with a structured way to communicate the emotional and psychological impact of their conditions. As a result, clinicians can gain a deeper understanding of how skin diseases affect their patients’ lives, potentially enhancing empathy and strengthening doctor–patient relationships. This awareness may also improve adherence to treatment plans, as patients feel heard and understood. Ultimately, the DLQI fosters a more comprehensive approach to dermatological care, emphasising the importance of addressing both physical and psychological aspects of disease management.

## Conclusion and recommendations

This study demonstrates that dermatological conditions significantly impact patients’ QoL to varying degrees across both sexes. Among the participants, eczema and acne were the most commonly encountered conditions. Researchers further identified nine cutaneous disorders that had the greatest impact on QoL: HS, cutaneous lupus, urticaria, alopecia, psoriasis, vitiligo, eczema, cutaneous malignancies and acne. Of these, HS, lupus and urticaria had the most substantial effect on patients’ well-being and life quality.

Nonetheless, more robust, further studies are still needed to draw more accurate conclusions. The DLQI assessed facets such as physical activity, emotional expression, professional functioning and social interactions. Integrating DLQI as a tool into daily practice could positively influence management strategies by dermatologists.

### Study limitations and quality improvement for future studies

This study was conducted at a single tertiary hospital, which limits the ability to generalise the findings about the QoL of all patients affected by the specified disease conditions. Additionally, the patients were not evenly distributed among the different disease conditions, making precise comparisons challenging. The unequal number of patients across various diagnostic categories may have introduced discrepancies in the results. Using the DLQI more efficiently would have to be in a longitudinal study setting, rather than a cross-sectional one as in our case. One also has to take a specific condition and follow the patient’s scores as an adjunct to treatment response.

## References

[1] Darlenski R, Kazandjieva J, Tsankov N. Skin barrier function: Morphological basis and regulatory mechanisms. J Clin Med. 2011;4(1):36–45.

[2] Pärna E, Aluoja A, Kingo K. Quality of life and emotional state in chronic skin disease. Acta DV. 2015;95(3):312–316. 10.2340/00015555-1920 24978135

[3] Vilar GN, Santos LA, Sobral Filho JF. Quality of life, self-esteem and psychosocial factors in adolescents with acne vulgaris. An Bras Dermato. 2015;90(5):622–629. 10.1590/abd1806-4841.201533726 PMC4631226 26560206

[4] Wong S-M, Baba R. Quality of life among Malaysian patients with vitiligo. Int J Dermatol. 2012;51(2):158–161. 10.1111/j.1365-4632.2011.04932.x 22250623

[5] Holm EA, Wulf HC, Stegmann H, Jemec GBE. Life quality assessment among patients with atopic eczema. Br J Dermatol. 2006;154(4):719–725. 10.1111/j.1365-2133.2005.07050.x 16536816

[6] Maksimovic N, Janković S, Marinković J, Sekulović LK, Živković Z, Spirić VT. Health-related quality of life in patients with atopic dermatitis. J Dermatol. 2011;39(1):42–47. 10.1111/j.1346-8138.2011.01295.x 22044078

[7] De Jager ME, Van De Kerkhof PCM, De Jong EMGJ, Seyger MMB. A cross-sectional study using the Children’s Dermatology Life Quality Index (CDLQI) in childhood psoriasis: Negative effect on quality of life and moderate correlation of CDLQI with severity scores. Br J Dermatol. 2010;163(5):1099–1101. 10.1111/j.1365-2133.2010.09993.x 20716218

[8] Szepietowski JC, Reich A. Quality of life and pruritus. In: Preedy VR, Watson RR, editors. Handbook of disease burdens and quality of life measures. New York: Springer, 2010; p. 2151–162.

[9] Myers EM, Perche PO, Jorizzo JL, Feldman SR. Reducing costs in atopic dermatitis. Dermatol Ther. 2022;35(11):e15849. 10.1111/dth.15849 36131640

[10] Yano C, Saeki H, Ishiji T, et al. Impact of disease severity on work productivity and activity impairment in Japanese patients with atopic dermatitis. J Dermatol. 2013;40(9):736–739. 10.1111/1346-8138.12220 23834561

[11] Agner T, Andersen KE, Brandao FM, et al. Hand eczema severity and quality of life: A cross-sectional, multicentre study of hand eczema patients. Contact Dermatitis. 2008;59(1):43–47. 10.1111/j.1600-0536.2008.01362.x 18537992

[12] Al-Otaibi HM, AlFurayh NA, AlNooh BM, Aljomah NA, Alqahtani SM. Quality of life assessment among patients suffering from different dermatological diseases. Saudi Med J. 2021;42(11):1195–1200. 10.15537/smj.2021.42.11.20210560 34732551 PMC9149728

[13] McKoy K. The importance of dermatology in global health [homepage on the Internet]. Burlington. [updated 2011; cited 2022 May 18]. Available from: https://files.ctctcdn.com/ded15bfa001/e46f16b0-8960-4f2c-8222-5d1b327e3e46.pdf

[14] Amer AA, Gao X-H. Quality of life in patients with vitiligo: An analysis of the dermatology life quality index outcome over the past two decades. Int J Dermatol. 2016;55(6):608–614. 10.1111/ijd.13198 26749040

[15] Ongenae K, Beelaert L, Van Geel N, Naeyaert J-M. Psychosocial effects of vitiligo. J Eur Acad Dermatol Venereol. 2006;20(1):1–8. 10.1111/j.1468-3083.2005.01369.x 16405601

[16] Lai YC, Yew YW, Kennedy C, Schwartz RA. Vitiligo and depression: A systematic review and meta-analysis of observational studies. Br J Dermatol. 2017;177(3):708–718. 10.1111/bjd.15199 27878819

[17] Mesinkovska N, King B, Mirmirani P, Ko J, Cassella J. Burden of illness in alopecia areata: A cross-sectional online survey study. J Investig Dermatol Symp Proc. 2020;20(1):S62–S68. 10.1016/j.jisp.2020.05.007 33099390

[18] Rapp SR, Feldman SR, Exum ML, Fleischer AB, Reboussin DM. Psoriasis causes as much disability as other major medical diseases. J Am Acad Dermatol. 1999;41:401–407. 10.1016/S0190-9622(99)70112-X 10459113

[19] Hong J, Koo B, Koo J. The psychosocial and occupational impact of chronic skin disease. Dermatol Ther. 2008;21(1):54–59. 10.1111/j.1529-8019.2008.00170.x 18318886

[20] Alavi A, Farzanfar D, Lee RK, Almutairi D. The contribution of malodour in quality of life of patients with Hidradenitis Suppurativa. J Cutan Med Surg. 2018;22(2):166–174. 10.1177/1203475417745826 29231053

[21] Alavi A, Anooshirvani N, Kim WB, Coutts P, Sibbald. Quality-of-life impairment in patients with Hidradenitis Suppurativa: A Canadian study. Am J Clin Dermatol. 2015;16(1):61–65. 10.1007/s40257-014-0105-5 25432664

[22] Ghodsi SZ, Chams-Davatchi C, Daneshpazhooh M, Valikhani M, Esmaili N. Quality of life and psycholuogical status of patients with pemphigus vulgaris using dermatology life quality index and general health questionnaires. J Dermatol. 2012;39(2):141–144. 10.1111/j.1346-8138.2011.01382.x 21967321

[23] Sanclemente G, Burgos C, Nova J, et al. The impact of skin diseases on quality of life: A multicenter study. Actas Dermosifiliogr. 2017;108(3):244–252. 10.1016/j.ad.2016.11.008 28063525

[24] Katugampola RP, Lewis VJ, Finlay AY. The dermatology life quality index: Assessing the efficacy of biological therapies for psoriasis. Br J Dermatol. 2007;156(5):945–950. 10.1111/j.1365-2133.2007.07817.x 17388922

[25] Steinsbekk S, Wichstrøm L, Stenseng F, Nesi J, Hygen BW, Skalická V. The impact of social media use on appearance self-esteem from childhood to adolescence – A 3-wave community study. Comput Human Behav. 2021;114:106528. 10.1016/j.chb.2020.106528

[26] Jiotsa B, Naccache B, Duval M, Rocher B, Grall-Bronnec M. Social media use and body image disorders: Association between frequency of comparing one’s own physical appearance to that of people being followed on social media and body dissatisfaction and drive for thinness. Int J Environ Res Public Health. 2021;18(6):2880. 10.3390/ijerph18062880 33799804 PMC8001450

[27] Hazarika N, Rajaprabha RK. Assessment of life quality index Among patients with acne vulgaris in a suburban population. Indian J Dermatol. 2016;61(2):163–168. 10.4103/0019-5154.177758 27057015 PMC4817440

[28] Finlay AY, Khan GK. Dermatology Life Quality Index (DLQI)—a simple practical measure for routine clinical use. Clin Exp Dermatol. 1994;19(3):210–216. 10.1111/j.1365-2230.1994.tb01167.x 8033378

[29] Abolfotouh MA, Al-Khowailed, Suliman, Al-Turaif, Al-Bluwi, Al-Kahtani. Quality of life in patients with skin diseases in Central Saudi Arabia. Int J Gen Med. 2012;5:633–642. 10.2147/IJGM.S33276 22866015 PMC3410718

[30] Vyas J, Johns JR, Abdelrazik Y, et al. The Dermatology Life Quality Index (DLQI) used as the benchmark in validation of 101 quality of life instruments: A systematic review. J Eur Acad Dermatol Venereol. 2025; 39(3):631–679. 10.1111/jdv.20321 39269008 PMC11851266

[31] Jobanputra R, Bachmann M. The effect of skin diseases on quality of life in patients from different social and ethnic groups in Cape Town, South Africa. Int J Dermatol. 2000;39(11):826–831. 10.1046/j.1365-4362.2000.00073.x 11123442

[32] Moodley N, Hoosen K, Dlova NC. Quality of life in patients with seborrhoeic dermatitis in KwaZulu-Natal, South Africa. S Afr Med J. 2016;106(5):428. 10.7196/SAMJ.2016.v106i5.10551

[33] Jordaan HF. Common skin and mucosal disorders in HIV and AIDS. SA Fam Pract. 2008;50(6):14–23. 10.1080/20786204.2008.10873772

[34] Schwartz RA. Cutaneous manifestations of HIV [homepage on the Internet]. Medscape-Dermatology Section. Available from: https://www.medscape.com

[35] Cedeno-Laurent F, Gómez-Flores M, Mendez N, et al. New insights into HIV-1-primary skin disorders. J Int AIDS Soc. 2011;14:5. 10.1186/1758-2652-14-5 21261982 PMC3037296

[36] Al-Hoqail IA. Impairment of quality of life among adults with skin disease in King Fahad Medical City, Saudi Arabia. J Fam Community Med. 2009;16(3):105–109. 10.4103/2230-8229.96527 PMC3377042 23012200

[37] Maruma F, Dlova N, Mofokeng TRP, Moloabi BC. Nevus of Hori in African patients: An entity that is most likely underdiagnosed in clinical practice. Int J Womens Dermatol. 2025;11(1):e190. 10.1097/JW9.0000000000000190 39764172 PMC11698270

[38] Dlova NC, Ajose F. Communication on the dangers and abuse of skin lighteners in Africa. Int J Dermatol. 2014;53(6):e335–e337. 10.1111/ijd.12221 23879291

[39] Dlova NC, Akintilo LO, Taylor SC. Prevalence of pigmentary disorders: A cross-sectional study in public hospitals in Durban, South Africa. Int J Womens Dermatol. 2019;5(5):345–348. 10.1016/j.ijwd.2019.07.002 31909155 PMC6938902

[40] Maruma F, Dlova N, Mofokeng TRP, Al-Niaimi F. Treatment outcomes for dermatosis papulosa Nigra using low-intensity electrodesiccation device in African patients. J Cosmet Dermatol. 2024;24(2):e16729. 10.1111/jocd.16729. 39663874 PMC11837235

[41] Silverberg JI, Gelfand JM, Margolis DJ, et al. Patient burden and quality of life in atopic dermatitis in US adults: A population-based cross-sectional study. Ann Allergy Asthma Immunol. 2018;121(3):340–347. 10.1016/j.anai.2018.07.006 30025911

[42] Khan JM, Rathore MU, Tahir M, Abbasi T. Dermatology life quality index in patients of psoriasis and its correlation with severity of disease. J Ayub Med Coll Abbottabad. 2020;32:64–67. 32468758

